# Epigenetic Mechanisms Contribute to the Expression of Immune Related Genes in the Livers of Dairy Cows Fed a High Concentrate Diet

**DOI:** 10.1371/journal.pone.0123942

**Published:** 2015-04-10

**Authors:** Guangjun Chang, Kai Zhang, Tianle Xu, Di Jin, Junfei Guo, Su Zhuang, Xiangzhen Shen

**Affiliations:** 1 College of Veterinary Medicine, Nanjing Agricultural University, Nanjing, P. R. China; 2 College of Animal Science and Technology, Nanjing Agricultural University, Nanjing, P. R. China; Institute of Farm Animal Genetics, GERMANY

## Abstract

**Purpose:**

Epigenetic modifications critically regulate the expression of immune-related genes in response to inflammatory stimuli. It has been extensively reported that a high concentrate (HC) diet can trigger systemic inflammation in dairy cows, yet it is unclear whether epigenetic regulation is involved in the expression of immune genes in the livers of dairy cows. This study aimed to investigate the impact of epigenetic modifications on the expression of immune-related genes.

**Experimental Design:**

In eight mid-lactating cows, we installed a rumen cannula and catheters of the portal and hepatic veins. Cows were randomly assigned to either the treatment group fed a high concentrate (HC) diet (60% concentrate + 40% forage, *n = 4*) or a control group fed a low concentrate (LC) diet (40% concentrate + 60% forage, *n = 4*).

**Results:**

After 10 weeks of feeding, the rumen pH was reduced, and levels of lipopolysaccharide (LPS) in the rumen, and portal and hepatic veins were notably increased in the HC group compared with the LC group. The expression levels of detected immune response-related genes, including Toll-like receptor 4 (TLR4), cytokines, chemokines, and acute phase proteins, were significantly up-regulated in the livers of cows fed a HC diet. Chromatin loosening at the promoter region of four candidate immune-related genes (TLR4, LPS-binding protein, haptoglobin, and serum amyloid A3) was elicited, and was strongly correlated with enhanced expression of these genes in the HC group. Demethylation at the promoter region of all four candidate immune-related genes was accompanied by chromatin decompaction.

**Conclusion:**

After HC diet feeding, LPS derived from the digestive tract translocated to the liver via the portal vein, enhancing hepatic immune gene expression. The up-regulation of these immune genes was mediated by epigenetic mechanisms, which involve chromatin remodeling and DNA methylation. Our findings suggest that modulating epigenetic mechanisms could provide novel ways to treat systemic inflammatory responses elicited by the feeding of a HC diet.

## Introduction

In the dairy industry, it is currently a common practice to feed a high concentrate (HC) diet to lactating cows to meet their energy requirements to support high milk production. Although this practice might increase short-term economic efficiency, it is not a beneficial practice for the health of cows. The effects of a HC diet on gastrointestinal tract or mammary gland health have received increasing attention [1–3]. However, to date, the effects of a HC diet on immune responses and liver metabolism have remained unclear, despite the extreme importance of these pathways in cow health and performance [4, 5]. The consumption of a HC diet causes a series of changes that involve a significant depression of pH and increased lipopolysacchrides (LPS) in the rumen and intestine, as well as the elevation of acute phase proteins (APPs), including LPS-binding protein (LBP), serum anyloid A (SAA), and haptoglobin (Hp), in the peripheral blood [6]. Changes in the blood concentrations of SAA and Hp, which have been reported in many studies, suggest that feeding a HC diet triggers an inflammatory response [7, 8]. Moreover, serum levels LBP are an indicator of systemic inflammation caused by circulating LPS that has translocated from the digestive tract [9]. Although many hazardous substances are released in the digestive tract and translocate into the bloodstream during feeding with a HC diet, LPS is thought to be the major contributor to systemic inflammation because most LPS is released by Gram-negative bacteria, which are the main bacterial community in the digestive tract of cows [10].

The liver is an important site where harmful substances (e.g., microbial products, LPS, and histamine) from the digestive tract encounter immune cells and stimulate the innate immune system, which is the first line of defense against invading pathogens. Innate immune cells, such as Kupffer cells, dendritic cells, and neutrophils, initiate and maintain hepatic inflammation by secreting cytokines [11]. A global gene expression analysis of liver tissues showed that the expression of cytokines and APPs was enhanced in experimental LPS intra-mammary gland infusion [12]. Moreover, consuming a HC corn straw diet caused a higher pro-inflammatory response in the mammary gland [3]. However, the effects of a HC diet on pro-inflammatory responses in the liver are largely unknown. Furthermore, consuming a HC diet leads to marked increases in LPS produced in the digestive tract and delivered directly to the liver via the portal vein [13]. These events trigger a liver inflammatory response via the TLR4-signaling pathway, resulting in the release of pro- and anti-inflammatory cytokines and APPs [14].

Excessive activation of the TLR4 signaling pathway by LPS induces endotoxin tolerance and cross-tolerance towards other pathogen-associated molecular patterns (PAMPs) [15]. Epigenetic mechanisms involved in chromatin remodeling are thought to mediate these phenomena [16–18]. A recent study conducted in dairy cows showed that feeding a HC corn straw diet elicited epigenetic alterations in mammary glands [19]. Additionally, *E*. *coli* mastitis has been reported to induce chromatin compaction at the αS1-casein promoter to shut down casein synthesis, and to trigger chromatin decompaction at the β-defensin LAP promoter to facilitate NF-κB recruitment, which drives enhanced gene expression [20, 21]. Although many advances have been made in recent years, the role of epigenetic mechanisms in the regulation of immune-related genes in the liver in response to gastrointestinal tract-derived LPS is less well-known. Therefore, we hypothesized that feeding a high concentrated diet to lactating cows results in the entry of excessive LPS into the liver via the portal vein, thereby enhancing the expression of hepatic immune related genes. We predicted that epigenetic mechanisms contribute to the up-regulated expression of innate immune genes. We tested this hypothesis by analyzing chromatin remodeling and DNA methylation at the promoter regions of immune-related genes. TLR4 and LBP were selected as parameters to evaluate activation of the TLR4 signaling cascade. Hp and SAA3 were chosen as model biomarkers to assess the acute phase reaction (APR). Finally, αS1-casein was used as an unmodulated control gene in the liver based on published data [20].

## Materials and Methods

### Ethics Statement

The experimental protocol was approved by the Animal Care Committee of Nanjing Agricultural University in accordance with the Guidelines for Experimental Animals of the Ministry of Science and Technology (2006, Beijing, China).

### Animal, Diets and Experimental Design

A total of eight multiparous mid-lactating Holstein dairy cows (average body weight, 512 ± 36 kg; average milk yield, 29.18 ± 3.23 kg, 1 to 2 months post partum) were used in this experiment and were randomly assigned to one of two groups. Cows were fed a HC (Forage: Concentrate = 4:6, *n = 4*) diet in the treatment group, and were fed low concentrated (LC, Forage: Concentrate = 6:4, *n = 4*) diet in the control group. Quarter milk samples of these cows were measured weekly two times during entire experimental period to be free from mastitis (somatic cells <100000 /mL). Before the beginning of formal experiment, all of cows were received LC diet for 4 weeks (as an adaption period) to ensure the similarity of metabolic status. Dietary ingredients and nutrient composition are shown in Table 1. The formal experiment lasted for 10 weeks. All experimental cows were housed in individual tie-stalls at the Dairy Farm of Nanjing Agricultural University (Nanjing, China). Animals were fed and milked three times daily at 04:00, 12:00, 20:00 h, and were allowed free access to fresh water. In the first week of the adaption period, cows were installed rumen cannula, and indwelt with hepatic and portal vein catheters. After surgery, cows were observed for 2 weeks during recovery from the surgery. Sterilized heparin saline (500 IU/mL) was used to prevent catheter blockage at daily 8-hour intervals until the end of the experiment.

**Table 1:** Ingredients and nutritional composition of the diets.

Item	Diets	
	Control	Treatment
**Ingredients (% of dry matter)**		
Chinese wildrye hay	10	15
Alfalfa hay	25	0
Corn silage	25	25
Corn	21.97	33
Pure wheat bran	2	3
Soybean meal	14.97	22.4
Limestone	0.4	0.6
Calcium phosphate dibasic	0.08	0.1
Salt	0.28	0.4
Premix ^a^	0.3	0.5
Forage: concentrate (F: C)	6: 4	4: 6
**Nutritional composition (% of dry matter)**		
NE, MJ/kg	6.32	6.52
CP, %	17.24	17.27
NDF, %	38.81	35.07
ADF, %	23.66	19.31
NFC, %	32.58	37.51
Ca, %	0.78	0.44
P, %	0.30	0.33

^a^ Premix provided: 3000, 1250, and 40 IU kg^-1^ of diet of vitamin A, D and E, and 6.25, 62.5, 62.5, 50, 0.25, 0.125, 0.125 mg kg^-1^ of diet of Cu, Fe, Zn, Mn, I, Se, Co, respectively.

NE, Net energy; CP, Crude Protein; NDF, Neutral Detergent Fiber; ADF, Acid Detergent Fiber; NFC, Non-Fiber Carbohydrate.

### Sample Collection

Milk yields were recorded daily. Rumen pH values were measured on the last 3 days at 2^th^, 4^th^, 6^th^, 8^th^, 10^th^ week. Rumen fluid and blood samples of the portal and hepatic veins were collected on the last 3 consecutive days of the 10^th^ week at 0 (15 min before feeding), 4, and 8 h after feeding. Rumen fluid samples were centrifuged at 10,000 ×*g* for 45 min, and then the supernatant was aspirated and passaged through a disposable 0.22-μm filter. The filtrate was collected in a sterile, depyrogenated glass tube (previously heated at 250°C for 2 h) and heated at 100°C for 30 min. Samples were cooled at room temperature for 15 min and stored at—20°C for subsequent LPS measurements. Plasma was isolated from blood samples by centrifugation at 1900 ×*g* at 4°C for 15 min, then was injected into pyrogen-free glass tubes and stored at—20°C for LPS measurements. The day after the collection of rumen fluid and blood samples was completed, liver tissue samples were obtained by punch biopsy under local anesthesia, immediately frozen in liquid nitrogen, and subsequently stored at—70°C, as previously described [12].

### LPS Measurements

LPS is the component of cell wall of Gram-negative bacteria that are the predominant bacterial group in the rumen. After the feeding of HC diet, rapid growth of Gram-negative bacteria can result in the shedding of LPS in the rumen in the early stage. Meanwhile, the rapid growth of those bacteria leads to the production of massive organic acid (for example VFA, lactic acid) repressing the rumen pH, which cause the death and cell lysis to release a large amount of free LPS [10]. In addition, when starch enters into colon or caecum, the same response will happen, and these changes (the reduction of pH and increase of LPS concentration in digestive tract) can cause the disruption of gastrointestinal barrier facilitating the translocation of LPS from digestive tract into circulation [1, 2].

The concentration of LPS was determined using a chromogenic endpoint assay (cat. CE64406, Chinese Horseshoe Crab Reagent Manufactory Co., Xiamen, China) with a minimum detection limit of 0.05 EU/mL (rumen liquid) or 0.01 EU/mL (plasma). Procedures were performed according to the manufacturer’s instructions, as described by Dong *et al*. [22].

### RNA Extraction and Real-Time Quantitative PCR (RT-qPCR)

RT-qPCR was performed using an ABI 7300 system (Applied Biosystems, Foster City, CA, USA) to determine the relative copy numbers of different mRNA transcripts. Liver samples were ground into a powder with a mortar under liquid nitrogen, and then total RNA was extracted in Trizol (Takara, Dalian, China) in accordance with the manufacturer’s protocol. For cDNA synthesis, 1.5 μg total RNA was prepared in a reverse transcription reaction (cat. RR036A, Takara) with one specific primer for each of the different mRNAs and oligo (dT) for all mRNAs. Then, cDNAs were purified using a purification kit (cat. D0033, Beyotime, Shanghai). RT-qPCR was run using gene-specific primer pairs to amplify cDNA target segments using SYBR Premix EX Taq kit (cat. DRR420A, Takara). The relative copy numbers of individual mRNA were calculated from a dilution series of 10^6^ to 10^2^ copies of the respective sequenced plasmids. All samples were assayed twice from two independent cDNA preparations. Reverse transcription and amplification primers are listed in S1 Table.

### Chromatin Precipitation and CHART-PCR (Restriction Enzyme Digestion, Purification of DNA, and q-PCR)

A 100-mg tissue sample was ground into a powder in liquid nitrogen and suspended in 3-mL pre-chilled resuspension buffer (RSB; 10 mM Tris [pH 8.0], 3-mM MgCl_2_, and 10-mM NaCl) that contained 0.5% Nonidet NP 40. A freshly prepared dilution (1/200) of a proteinase inhibitor cocktail (Roche) and phenyl-methyl-sulfonyl-fluoride (PMSF; 1 mM) were added immediately prior to use. After incubation for 5 min on ice, the tissue powder was homogenized using a dounce homogenizer (Sigma). The liquid was filtered through sterilized glass wool into a pre-cooled 15-mL Eppendorf tube, then the filtrate was centrifuged at 1000 *×g* for 10 min at 4°C to pellet the nuclei. Pellets were washed once in RSB buffer containing 1 mM β-mercaptoethanol and then were transferred into a pre-cooled 1.5-mL Eppendorf vial. Nuclei were pelleted again (1000 *×g*, 5 min, 4°C) and re-suspended in 100-μL RSB buffer containing 50% glycerol and stored at—20°C.

Chromatin compaction was measured using the Chromatin Accessibility by Real-time PCR assay (CHART) [23], as described Vanselow *et al* [20] with minor modifications. Briefly, 4-μL nuclear suspension was added to 46-μL restriction digest buffer supplemented with 20-U restriction enzyme and proteinase inhibitors (see above), and mixtures were incubated at 37°C for 30 min. A control sample was prepared by omitting the enzyme and treating it similarly. Subsequently, proteins were digested (56°C, 2 h) with proteinase K (0.5 mg/mL in 50 mM Tris [pH 8.0], 0.15 M EDTA, 0.1 M NaCl, 6 mM DTT, and 1% SDS). DNA was purified using a purification kit (cat. D0033, Beyotime) and the DNA concentration was measured using a Nanodrop spectrophotometer. The restriction enzymes used to determine the amount of chromatin compaction at the different promoters were M*ae*I (for TLR4, LBP, and SAA3) and D*de*I (for αS1-casein and Hp). It has been reported that αS1-casein encoding gene would be expressed to a considerable amount in the liver of cows suffering *E*.*coli* mastitis, and the degree of chromatin compaction at its promoter region had no difference in the liver between infectied cows and control cows [20, 24]. Hence, this gene was treated as unregulated control gene to highlight the sensitivity of immune relevant genes (TLR4, LBP, Hp and SAA3) to chromatin remodeling in the present study.

The quantity of undigested target DNA was measured by real-time PCR (ABI 7300), similarly to the protocol described above for RT-qPCR. Primer sequences are listed in S2 Table. The degree of compaction was represented as the fraction of the copy numbers determined from the digested *vs* undigested control samples.

### Methylation Assay of Target Gene Promoters

Genomic DNA was isolated from liver tissues and analyzed by Methyl-Profiler DNA Methylation qPCR Assays with some modifications [25, 26]. This method exploits the theory that H*pa*II digestion can be blocked by the methylation of C nucleotides in CpG dinucleotides, whereas its isoschizomer M*sp*I is not blocked. Purified genomic DNA was predigested with E*co*RI to facilitate accurate aliquoting. Subsequently, 3-μg purified DNA was distributed and digested with H*pa*II or M*sp*I (10 U, 37°C, 2 h), or was similarly treated without enzyme addition as a control. Subsequently, DNA was re-purified and re-quantified again and then 200 ng (equivalent of ~6000 gene copies) was used to determine the remaining DNA copies for the qPCR assays. Primer sequences are listed in S3 Table. The degree of methylation was calculated based on the difference between the ratio of copy numbers obtained after H*pa*II or M*sp*I digestion, and each relative value is presented compared with the value of the undigested control (set as 100% of input DNA).

### Western Blotting

Total proteins were extracted from smashed liver tissue with RIPA Lysis Buffer (cat. SN338, Sunshine Biotechnology Co., Nanjing, China). Protein concentrations were determined using the BCA assay (Pierce, Rockford, IL, USA). A total of 50-μg protein extract from each sample was subjected to electrophoresis on a 7.5% SDS–PAGE gel, and separated proteins were transferred onto nitrocellulose membranes (Bio Trace, Pall Co., Port Washington, NY, USA). Western blotting analysis of TLR4 (cat. sc-293072, Santa Cruz Biotechnology, Santa Cruz, CA, USA, 1:200) was carried out with the primary antibody and a corresponding HRP-conjugated secondary antibody. Additionally, β-actin (KC-5A08, Kang Chen Bio-tech, Shanghai, China, 1:5000) was used as a reference protein for normalization purposes in western blotting analysis. Then, blots were washed and detected by enhanced chemiluminescence (ECL) using the LumiGlo substrate (Super Signal West Pico Trial Kit, Pierce). ECL signals were recorded using an imaging system (Bio-Rad, Hercules, CA, USA) and analyzed with Quantity One software (Bio-Rad). The intensity of TLR4 protein bands are presented as the relative fold-change compared to the average value of the control group.

### Statistical Analysis

The mixed procedure of SAS (SAS version 9.2, SAS Institute Inc.) was used to analyze LPS concentrations with a repeated measures design. The effects of treatment and time were classified as fixed factors. The effects of cows were considered randomly. The amount of time within treatments and cows were considered as repeated measurements, and compound symmetry (CS) was used as the type of covariance. Analysis of rumen pH values, milk yields, selected mRNA expression levels, the degree of chromatin compaction, the percentage of promoter methylation, and TLR4 protein expression was performed using the ANOVA package of SAS. The coefficient correlations between the mRNA expression levels and the degree of chromatin compaction, as well as between the degree of chromatin compaction and the percentages of promoter methylation were analyzed using Pearson’s model in SAS. Significant differences that were detected were defined at two levels as follows: *p*<0.05 (significant) or *p*<0.01 (highly significant).

## Results

### Rumen pH Values and Milk Yields

Feeding a HC diet to cows linearly reduced the rumen pH in the treatment group compared with that in control group throughout the experimental period (*p*<0.01), and pH values were recorded from the 4^th^ week to the end of this experiment (Table 2). Milk yields was increased in first 4 weeks, and then gradually decreased in subsequent 6 weeks in the treatment group compared with that in the control group and obtained significantly difference at 10^th^ week (*p*<0.01, Table 2) between treatment and control groups.

**Table 2:** Rumen pH values and milk yields of cows from treatment and control groups.

Item	pH Value		Milk Yield	
	Control ^a^	Treatment ^a^	Control ^a^	Treatment ^a^
2^th^ Week	6.55 ± 0.03	6.48 ± 0.03	25.45 ± 1.07	25.28 ± 1.23
4^th^ Week	6.45 ± 0.04	6.18 ± 0.05**	25.90 ± 1.04	26.16 ± 1.22
6^th^ Week	6.42 ± 0.04	5.92 ± 0.06**	25.61 ± 1.51	23.23 ± 1.70
8^th^ Week	6.30 ± 0.03	5.71 ± 0.05**	24.91 ± 2.38	21.82 ± 1.79
10^th^ Week	6.38 ± 0.04	5.62 ± 0.05**	25.11 ± 0.68	20.12 ± 0.81**

^a^ Values are mean ± SEM

*p<0.05

**p<0.01 *vs* control.

### LPS Concentrations in the Rumen, Portal, and Hepatic Veins

LPS concentrations in rumen fluid were higher in the treatment group than that in the control group (*P* = 0.04), and the highest concentration of rumen LPS was observed at 4 h after feeding in the treatment group. The kinetic effects and temporal interactions with treatment on rumen LPS were not significant (Table 3). The concentrations of LPS in the portal (*p*<0.01) and hepatic (*p*<0.01) veins were significantly increased in the treatment group compared with those in the control group. The LPS concentrations in the portal and hepatic veins were both highest 4 h after feeding in the treatment group. The effects of treatment × time on LPS concentrations in the portal vein (*p* = 0.02) were significant, but were not in the hepatic vein (*p* = 0.15). The effects of time on LPS concentrations in the portal (*p* = 0.07) and hepatic (*p* = 0.24) veins were not significantly different between the treatment and control groups (Table 3).

**Table 3:** LPS concentrations in the rumen, and portal and hepatic veins of cows from treatment and control groups.

LPS Conc.	Control ^a^	Treatment ^a^	Tr, *p*	Ti, *p*	Tr × Ti, *p*
Rumen^#^ (EU/mL)					
0 h	3.79 ± 0.77	17.01 ± 0.61	0.04	0.85	0.06
4 h	6.04 ± 0.71	20.69 ± 0.62			
8 h	6.15 ± 0.15	18.03 ± 0.53			
Portal Vein (EU/mL)					
0 h	0.31 ± 0.05	0.88 ± 0.05	<0.01	0.07	0.02
4 h	0.28 ± 0.02	1.13 ± 0.08			
8 h	0.29 ± 0.04	0.81 ± 0.04			
Hepatic Vein (EU/mL)					
0 h	0.19 ± 0.05	0.58 ± 0.09	<0.01	0.24	0.15
4 h	0.13 ± 0.02	0.74 ± 0.06			
8 h	0.12 ± 0.09	0.53 ± 0.09			

^a^ Values are mean ± SEM

^#^ [×10^4^]

Tr, Treatment; Ti, Time; *p*, significant level.

### The Expression of Immune-Related Genes in the Liver

The expression levels of immune genes, encoding cytokines, chemokines, APPs, and TLR4, were up-regulated in the liver of the treated cows compared with control cows (Table 4). We found that the expression of the pro-inflammatory cytokines IL-1α, IL-1β, and TNF-α were similarly increased by three-fold, whereas the expression levels of IL-6 were elevated by 47-fold in the treatment group compared with those in the control group. The expression levels of acute phase proteins (LBP, SAA3, and Hp) showed highly significant differences (*p*<0.01) between the treatment and control groups, but the fold changes in expression varied. The expression levels of LBP, SAA3, and Hp were increased by 10.32-, 8.64-, and 3.97-fold, respectively, in the treatment group compared with those in the control group. TLR4, the specific receptor for LPS, was significantly elevated by 4.48-fold (*p*<0.05). Additionally, the expression of αS1-casein was not significantly different between the treatment and control groups, which we used as a control gene that was unmodulated for subsequent epigenetic analyses.

**Table 4:** The levels of mRNA expression (relative copy number) in the livers of cows from treatment and control groups.

Gene	Comment	Control ^a^	Treatment ^a^	Fold changes
TLR4	LPS recognition receptor	931 ± 90	4168 ± 1273*	4.48
LBP^&^	LPS binding protein	6.13 ± 3.22	63.21 ± 9.88**	10.32
IL-1A	Cytokine	292 ± 40	1144 ± 358*	3.92
IL-1B^&^	Cytokine	1.00 ± 0.16	3.06 ± 0.90*	3.06
IL-6	Chemokine	61 ± 16	2873 ± 1115*	47.10
TNF-α	Cytokine	2508 ± 524	8786 ± 1327**	3.50
IL-8	Cytokine	1041 ± 254	3895 ± 1129*	3.74
IL-10	Cytokine	656 ± 131	2435 ± 412**	3.71
CCL5^&^	Chemokine	0.36 ± 0.21	2.31 ± 0.27**	6.48
CCL20	Chemokine	253 ± 74	1317 ± 297**	5.21
SAA3^&^	APP^$^	2.88 ± 1.65	24.86 ± 3.62**	8.64
HP^&^	APP^$^	46.13 ± 16.05	183.21 ± 28.03**	3.97
αS1-casein^&^	Casein	54.61 ± 13.77	58.98 ± 12.35	1.08

^a^ Values are mean ± SEM

^&^copy number[×10^4^]

^$^acute phase protein (APP)

*p <0.05

**p <0.01 *vs* control.

### A Map of the Binding Sites for Relevant Transcription Factors in Promoters of Candidate Genes

**Fig 1:**
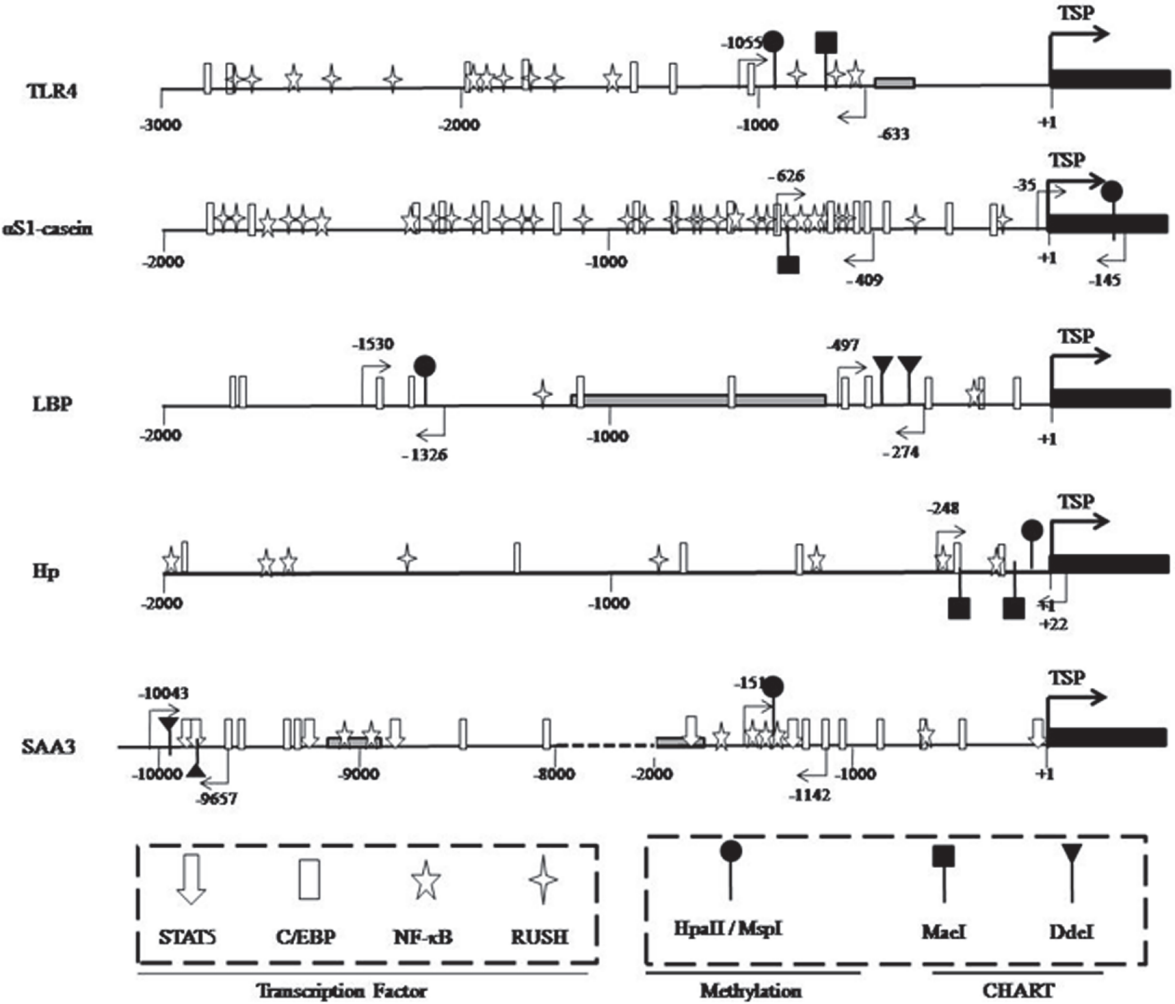
A map of the target promoter region of candidate genes and the distribution of relevant transcription factor binding sites in the corresponding promoter regions of those genes. Numbers refer to the transcriptional start position (TSP, +1), indicated by bold black arrows, and the 5’-position of exon 1 (black box). The gray boxes in the TLR4, Hp, and αS1-casein promoter regions indicate repeat elements. The position of the various transcription factors and restriction enzyme recognition sites are indicated by the respective symbols. The positions of primers used for CHART-PCR and the methylation assays are denoted by light black arrows. The promoter structure of αS1-casein has been described by Vanselow *et al*. The TSP of the SAA3 promoter was reported by Marilynn *et al*. The target promoter regions of candidate genes were identified by BLAST analyses as DNA-sequences that are 5’-upstream of the mRNA sequence deposited in the following NCBI files: NM_174198 (TLR4), NM_001038674 (LBP), NM_001040470 (Hp), and NW_003104637 (SAA3).

The target promoter sequences of all candidate genes, including TLR4, LBP, Hp, SAA3, and αS1-casein, were analyzed using the MatInspector program (Genomatix, online tools) to search for potential binding sites for relevant transcription factors. Subsequently, a map of all target gene promoters was prepared to show the positions of relevant binding sites of transcription factors and restriction enzymes that are involved in CHART and methylation assays, as well as the positions of primer pairs associated with those assays (Fig 1).

### Chromatin Remodeling of Immune Gene Promoters

**Fig 2:**
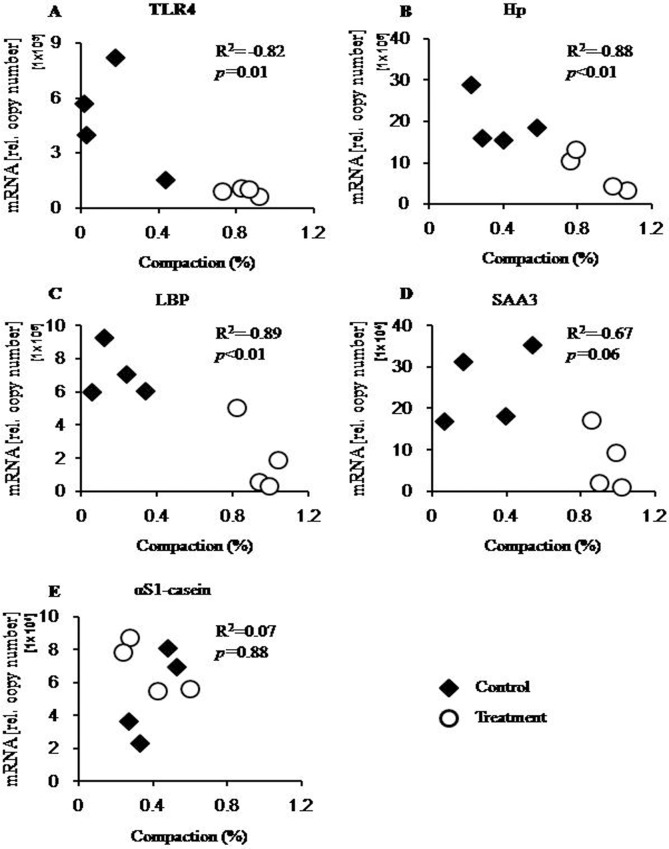
The correlation between chromatin compaction and mRNA expression. The graph shows the individual degrees of chromatin compaction (axis of abscissa) compared with the relevant mRNA copy numbers (axis of ordinate) for each respective gene, which are presented in panels A–E. Black rhombs, treated cows fed a HC diet; white circles, control cows fed a LC diet; R^2^, coefficient of correlation; *p*, significance of correlation.

Loosening of the chromatin at the promoter region of the four immune candidate genes was observed in the treatment group (Table 5). The average degree of chromatin compaction in the liver samples from the treated cows (0.26 ± 0.04) was much lower than that in samples from the control cows (0.91 ± 0.02), and this difference was highly significant (*p*<0.01). By contrast, the average degree of chromatin compaction at the aS1-casein promoter was very similar in liver samples from both groups (0.40 ± 0.06 *vs* 0.39 ± 0.08; treatment *vs* control, respectively). Moreover, plotting the individual degree of chromatin compaction against the relative mRNA copy numbers showed a distinct interdependence of both parameters for the four immune genes (Fig 2). The coefficients of correlation (R^2^) for TLR4 (R^2^ = -0.82, *p* = 0.013), LBP (R^2^ = -0.89, *p*<0.01), and Hp (R^2^ = -0.88, *p*<0.01) were all robust and significant. While the R^2^ of SAA3 was lower than the others, it still showed a significant correlation (R^2^ = -0.67, *p* = 0.05). Additionally, there was no significant correlation (R^2^ = 0.07, *p* = 0.88) between the degree of chromatin compaction at the aS1-casein promoter and the relative mRNA copy numbers of this gene.

**Table 5:** A comparison of chromatin compaction in the liver of cows between treatment and control groups.

Gene	Control ^a^	Treatment ^a^
TLR4	0.84 ± 0.04	0.17 ± 0.10**
LBP	0.95 ± 0.05	0.19 ± 0.06**
Hp	0.90 ± 0.08	0.38 ± 0.08**
SAA3	0.94 ± 0.04	0.30 ± 0.11**
***Mean (candidate immune genes)***	0.91 ± 0.02	0.26 ± 0.04**
αS1-casein	0.39 ± 0.08	0.40 ± 0.06

^a^ Values are mean ± SEM

*p < 0.05

**p < 0.01 *vs* control.

### Promoter Demethylation for Candidate Immune Genes

**Fig 3:**
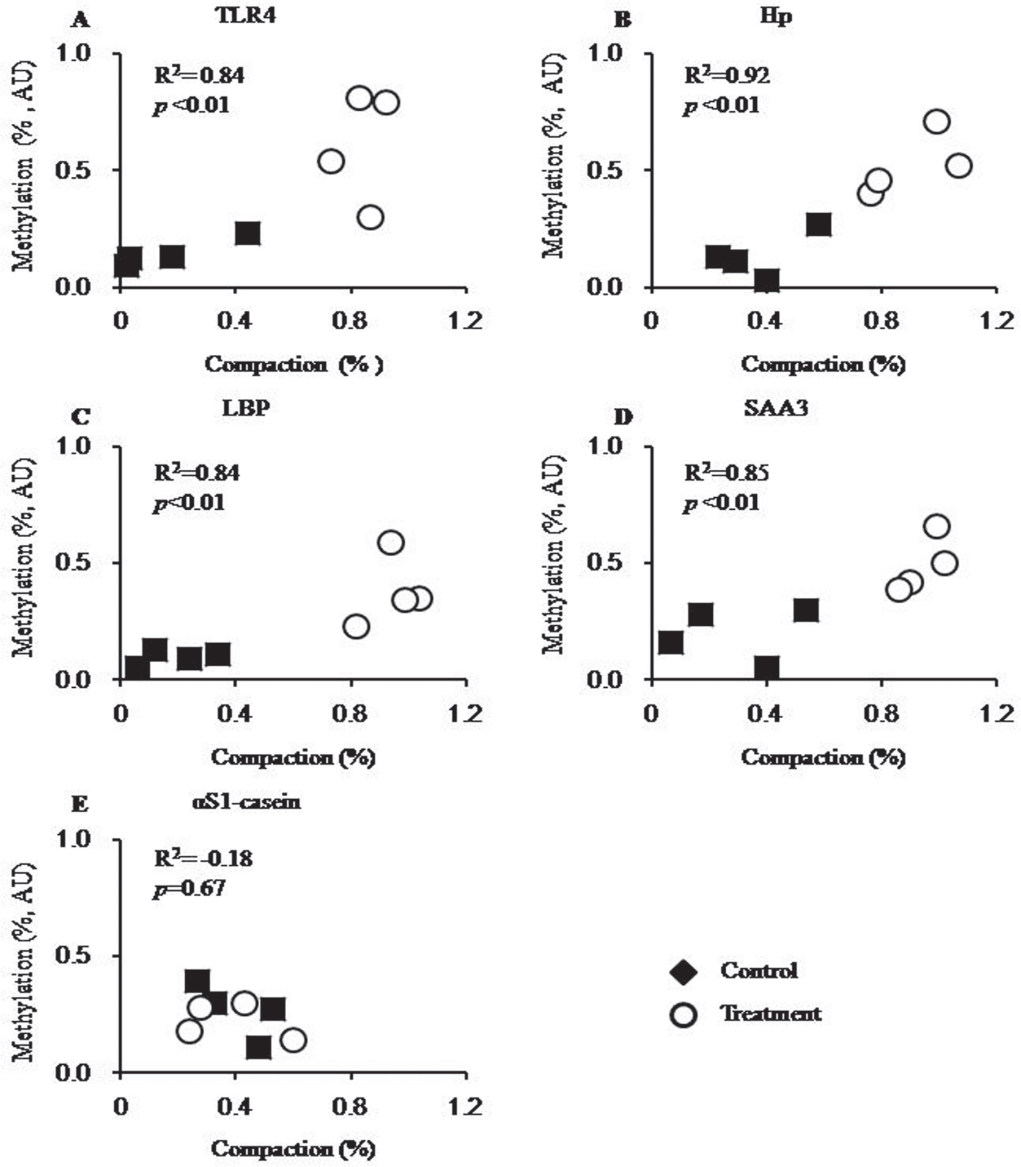
The correlation between the degree of chromatin compaction and the percentage of promoter methylation. The individual degrees of chromatin compaction (axis of abscissa) are plotted against the percentages of promoter methylation for the respective genes, as shown in panels A–E. Black squares, treated cows fed a HC diet; white circles, control cows fed a LC diet; R^2^, coefficient of correlation; *p*, significance of correlation.

A single H*pa*II/M*sp*I restriction site in the promoter region of these candidate genes (Fig 1) was used to probe for an eventual association of CpG demethylation with chromatin loosening. This site in all immune candidate gene promoters was hypomethylated in liver samples obtained from cows in the treatment group (Fig 3). The average degree of promoter methylation in liver samples from treatment group cows (0.14 ± 0.02) was significantly lower than that in liver samplers from control group cows (0.52 ± 0.01; *p*<0.01; Table 6). Compared with these immune genes, there was no significant difference in αS1-casein promoter methylation between the treatment and control groups, and the average degree of αS1-casein promoter methylation in the samples from both groups was much more similar (0.27 ± 0.06 *vs* 0.22 ± 0.04; treatment *vs* controls, respectively).

**Table 6:** A comparison of promoter methylation in the liver of cows from treatment and control groups.

Gene	Control ^a^	Treatment ^a^
TLR4	0.61 ± 0.12	0.14 ± 0.03**
LBP	0.38 ± 0.08	0.10 ± 0.02*
Hp	0.52 ± 0.07	0.14 ± 0.05**
SAA3	0.49 ± 0.06	0.20 ± 0.06*
***Mean (candidate immune genes)***	0.50 ± 0.04	0.14 ± 0.02**
αS1-casein	0.22 ± 0.04	0.27 ± 0.06

^a^ Values are mean ± SEM

*p < 0.05

**p < 0.01 *vs* control.

Plotting the individual degree of chromatin compaction against the degree of promoter methylation showed a strong relationship between these two parameters for the immune candidate genes. Our data analysis of promoter methylation showed a significant correlation for the four candidate immune genes as follows: TLR4 (R^2^ = 0.82, *p* = 0.01), LBP (R^2^ = 0.89, *p*<0.01), Hp (R^2^ = 0.92, *p*<0.01), and SAA3 (R^2^ = 0.85, *p*<0.01; Fig 3). By contrast, no significant correlation (R^2^ = -0.49, *p* = 0.22) was detected between the degree of chromatin compaction and the degree of promoter methylation for the αS1-casein encoding gene (Fig 3).

### TLR4 Protein Expression

**Fig 4:**
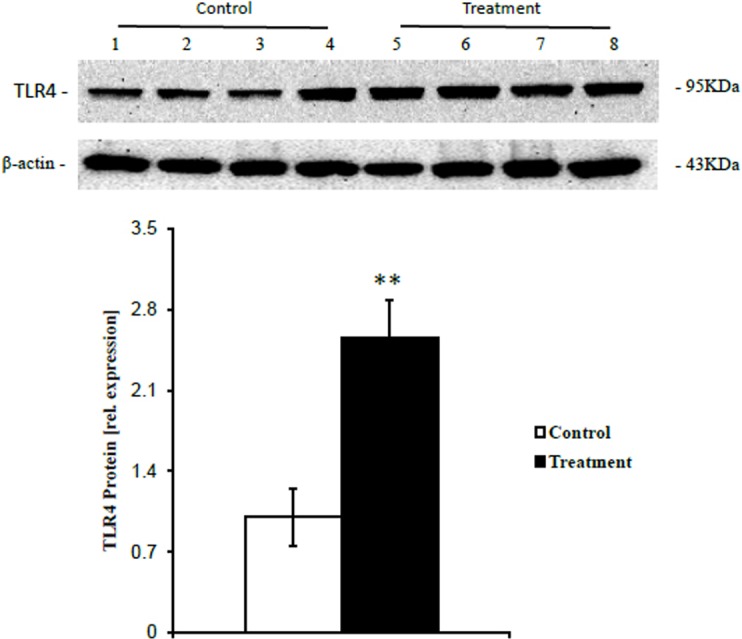
The expression of TLR4 protein in the livers of cows fed two different diets. The average relative TLR4 protein expression levels compared with reference β-actin protein levels is presented. Black filled bar, treatment group fed a HC diet (n = 4, mean ± SEM); white filled bar, control group fed a LC diet (n = 4, mean ± SEM). The significance of the changes in TLR4 protein expression levels is indicated (*p<0.05, **p<0.01).

Western blotting analysis showed that the expression of TLR4 protein, which initiates the TLR4-dependent innate immune signaling pathway, was significantly elevated (*p*<0.01) in liver samples from treatment group cows compared with control group cows (Fig 4).

## Discussion

Our study shows that during the feeding of HC diet, the up-regulated expression of immune related genes could be triggered by the increased translocation of LPS into the liver. Epigenetic modifications were involved in the regulation of the expression of immune related genes. These findings yield new insights into the contributions of epigenetic mechanism to hepatic inflammatory responses in dairy cows.

In recent decades, the practice of feeding HC diets to cows has been extensively applied to increase milk yields, thereby improving cost-efficiency. However, the consumption of a HC diet is harmful to the health of dairy cows [27]. Based on practical experience, two different diets, HC and LC diets, were designed and used in our present study. The main difference between these two diets was the different contents of NDF, ADF, and NFC, as indicated. The percentage of NDF and ADF in the nutritional composition was lower, but NFC was higher in the treatment diet than that in the control diet. Many studies have indicated that feeding this easily fermentable diet to cows is associated with a low rumen pH [8, 9]. In accord with previous studies, in our present study the rumen pH of cows fed a HC diet decreased linearly compared with that of cows fed a LC diet. A reduction in rumen pH during HC diet feeding caused the collapse of Gram-negative bacteria populations and resulted in increased free rumen LPS concentrations. In the study of Khafipour *et al*., replacing 21% of the dry matter of the total mixed ratio with pellets containing 50% wheat and 50% barley results in reduced milk yields and the percentage of milk fat, accompanied by reductions in rumen pH. The results of our study showed that milk yields increased in the first 4 weeks, and then declined in the subsequent 6 weeks in cows fed a HC diet compared with cows fed a LC diet, although the levels of crude protein and net energy were similar between these two diets. One reason for this finding could be that the buffering capacity of the rumen might counteract the decline of rumen pH in cows fed a HC diet in the short-term, whereas the extent of rumen pH depression could have exceeded the buffering capacity of the rumen, resulting in the decline of milk yield. Furthermore, in this and previous studies [6, 28], feeding of a HC diet induced the massive release of LPS in the rumen, which could trigger a local or systemic inflammatory response after the translocation of LPS into the bloodstream. In this setting, more nutrients would be used to sustain the inflammatory response, thereby reducing nutrient availability for milk synthesis. In a recent study, feeding a HC corn straw diet to cows elicited a pro-inflammatory response in the mammary gland and reduced milk yields [3]. Circulating LPS has been reported to depress the activity of key enzymes in milk component synthesis, such as acetyl-CoA carboxylase, lipoprotein lipase, and fatty acid synthase, and led to lesions in the mammary epithelia [10], thereby resulting in reduced milk yield.

Our data also showed that concentrations of LPS in the portal and hepatic veins were higher in cows fed a HC diet than that in cows fed a LC diet. Taking the concentrations of LPS in the rumen and portal veins into consideration, our findings indicated that a HC diet induced the translocation of LPS from the digestive tract into the bloodstream, and the amount of LPS delivered directly into the liver via the portal vein was higher in cows fed a HC diet compared with that in cows fed a LC diet. Recent experiments showed that during HC diet feeding, LPS produced in the digestive tract can contribute to local inflammatory responses in the rumen and intestine, thereby resulting in injury of the gastrointestinal barrier and facilitating the translocation of LPS and other hazardous substances into the circulating bloodstream [1, 29]. Although severe local inflammation in peripheral tissues might result in pro- and anti- inflammatory responses in the liver [12, 30], LPS in circulating blood, especially in the portal vein, is thought to be a major inducer of liver inflammation. Our measurements of the relative mRNA copy numbers by RT-qPCR confirmed that all of the liver samples from cows fed a HC diet showed signs of inflammation. Additionally, circulating LPS bound to LBP can be transferred to TLR4 located on the surface of innate immune cells, especially Kupffer cells (liver macrophages), to activate the intracellular TLR4-dependent signaling pathway, which results in the secretion of cytokines and chemokines [31]. We found that the feeding of a HC diet significantly increased the levels of mRNA encoding TLR4 and LBP, as well as the levels of TLR4 protein in the liver. These findings suggested that the increased translocation of LPS into the liver strongly activated the TLR4-dependent signaling pathway, which subsequently induced increased expression levels of pro- and anti-inflammatory cytokines (IL-1α, IL-1β, IL-6, and IL-10) and chemokines (IL-8, CCL5, and CCL20); such pro-inflammatory cytokines further induced the secretion of APPs by hepatocytes [32, 33]. Overall, our findings indicate that the liver of cows fed a HC diet are inflamed because of the excessive translocation of LPS into the liver via the portal vein.

The transcriptional start position (TSP) of each gene is a site that identifies the promoter region. The TSP of the gene encoding TLR4 was identified based on the results of the 5’-RACE experiment that we performed in a previous study, which showed that the predicted exon 1 in the database also acts as exon 1 in the liver. So, the 5’-adjacent region was considered to be its promoter. In the study of Marilynn *et al*., the TSP of SAA3 was also described [34]. For the genes encoding LBP and Hp, we identified that the TSP of both genes by blasting genomic DNA sequences with the respective mRNA sequences. The results of a MatInspector analysis of the promoters of LBP and Hp showed that both promoters indeed featured highly-conserved binding sites for the NF-κB (LBP) and C/EBP (LBP and Hp) transcription factors, which had previously been reported to be associated with expression of these genes [35, 36]. Moreover, an analysis of the promoter of the gene encoding aS1-casein was previously reported [20]. Finally, we confirmed the core promoter region of these genes, according to published data and our MatInspector analysis, and used them for further analysis by CHART and methylation.

Chromatin accessibility PCR was used to analyze the status of chromatin [23]. The local chromatin structure at promoters is a main factor that is highly associated with the amount of gene transcription. Our analysis of four immune related candidate genes firmly indicated that the loosening of chromatin at the promoter target region was significantly correlated with enhanced gene expression of immune genes. A previous study clearly demonstrated that the up-regulation of LAP expression could be attributed to chromatin decompaction at the promoter of bovine LAP, which facilitates NF-κB recruitment [21]. Additionally, a scatter analysis of the individual values of the expression of each candidate immune gene clearly demonstrated that chromatin loosening might be only one factor among several determinants that is important for inducing increased gene expression. Apart from chromatin remodeling, histone and DNA modifications are also necessary to create a proper chromatin environment to enhance or depress gene expression [37]. Furthermore, our data showed that the degree of chromatin compaction at the αS1-casein promoter was not different between the two groups fed different diets. Moreover, no significant correlation was detected between the degree of chromatin compaction at the αS1-casein promoter and its mRNA expression levels. These results showed that chromatin remodeling is highly related and specific to the expression level of immune-related genes. It is well-known that during sepsis, a severe illness caused by excessive endotoxin levels, chromatin remodeling is a key mechanism that drives the induction of endotoxin tolerance, which is induced to control excessive LPS-induced inflammation [18]. Herein, our study indicated that milder inflammation, which could be induced by circulating LPS derived from the digestive tract, could also trigger similar epigenetic mechanisms.

DNA methylation has been extensively studied and is thought to be one of the epigenetic mechanisms that can trigger chromatin compaction via methyl-CpG binding protein 2 (MeCP2) [38, 39]. Previously, the regulation of TLR2 and TLR4 expression in inflammation was shown to be associated with DNA methylation [40, 41]. In this present study, single H*pa*II restriction sites at the promoters of five candidate genes were analyzed to characterize the interdependence of CpG methylation and chromatin remodeling. Our data demonstrated that this site at the promoter of four immune relevant candidate genes was indeed hypomethylated in liver samples from cows fed a HC diet compared with those in liver samples from cows fed a LC diet. This finding indicated that after feeding a HC diet to cows, demethylation at the promoter of immune-related genes resulted in the loosening of chromatin at the corresponding promoter regions of those genes, thereby enhancing the expression of these genes. An earlier study showed that promoter hypomethylation can elicit histone hyperacetylation and release of the transcriptional repressor MeCP2, resulting in the activation of transcription [42]. Several studies have been preformed to examine the roles of epigenetic markers, such as DNA methylation and histone acetylation, on the regulation of LPS-induced innate immune gene expression [43, 44]. Additionally, the increased levels of LPS in the bloodstream of dairy cows fed a high proportion of concentrated diet have been well documented [3, 6]. In this present study, the content of LPS in the portal vein was significantly increased during feeding of a HC diet. Hence, the loosening of chromatin and hypomethylation at the promoter of immune-related genes in the liver of cows fed a HC diet could be attributed to increased translocation of LPS into the liver via the portal vein. Furthermore, the difference in the percentage of methylation at the αS1-casein promoter between the cows fed the two different diets was not significant. Thus, these data indicated that the expression of αS1-casein in the liver was not affected by the increased translocation of LPS into the liver, which highlights the sensitivity of immune-related genes to LPS.

Herein, we focused on the contribution of chromatin decompaction and DNA demethylation to the expression of immune genes. Further studies will be needed to elucidate the epigenetic regulation of histone modification and microRNAs. Considering all of the diverse modulators of chromatin remodeling, such as histone deacetylases [45, 46] or vitamin D_3_ [47, 48], these data might yield novel ways to regulate systemic inflammation, which can be induced by LPS that translocates from the digestive tract into the blood, and to improve high milk performance during productive practice of feeding a HC diet to cows.

In summary, our data indicate that feeding a HC diet to cows depresses the rumen pH value, results in the increase of LPS concentration in rumen. It has been previously reported that the feeding of HC diet caused the disruption of gastrointestinal barrier [1], this contributes to the translocation of LPS from digestive tract into portal vein. Hence, the increased translocation of LPS into the liver via the portal vein enhances the hepatic expression of immune-related genes, which are mediated by epigenetic mechanisms involving in chromatin decompacion and DNA demethylation. Our data confirmedly showed that chromatin decompaction and DNA demethylation in relevant areas of the promoter of candidate immune genes are strongly correlated with the enhanced expression of those immune genes. These findings demonstrate that epigenetic mechanisms contribute to the expression of immune-related genes in the livers of dairy cows fed a HC diet.

## Supporting Information

S1 Fig Gel images of promoter methylation. (TIF)

S1 Table Primers for RT-qPCR. (DOCX)

S2 Table Primers for CHART-PCR. (DOCX)

S3 Table Primers for methylation analysis. (DOCX)
