# Supplementary figures and images for: Epigenetic Mechanisms Contribute to the Expression of Immune Related Genes in the Livers of Dairy Cows Fed a High Concentrate Diet
Source: PLoS One. 2015 Apr 10;10(4):e0123942. doi: 10.1371/journal.pone.0123942 (PMC4393131; doi:10.1371/journal.pone.0123942)

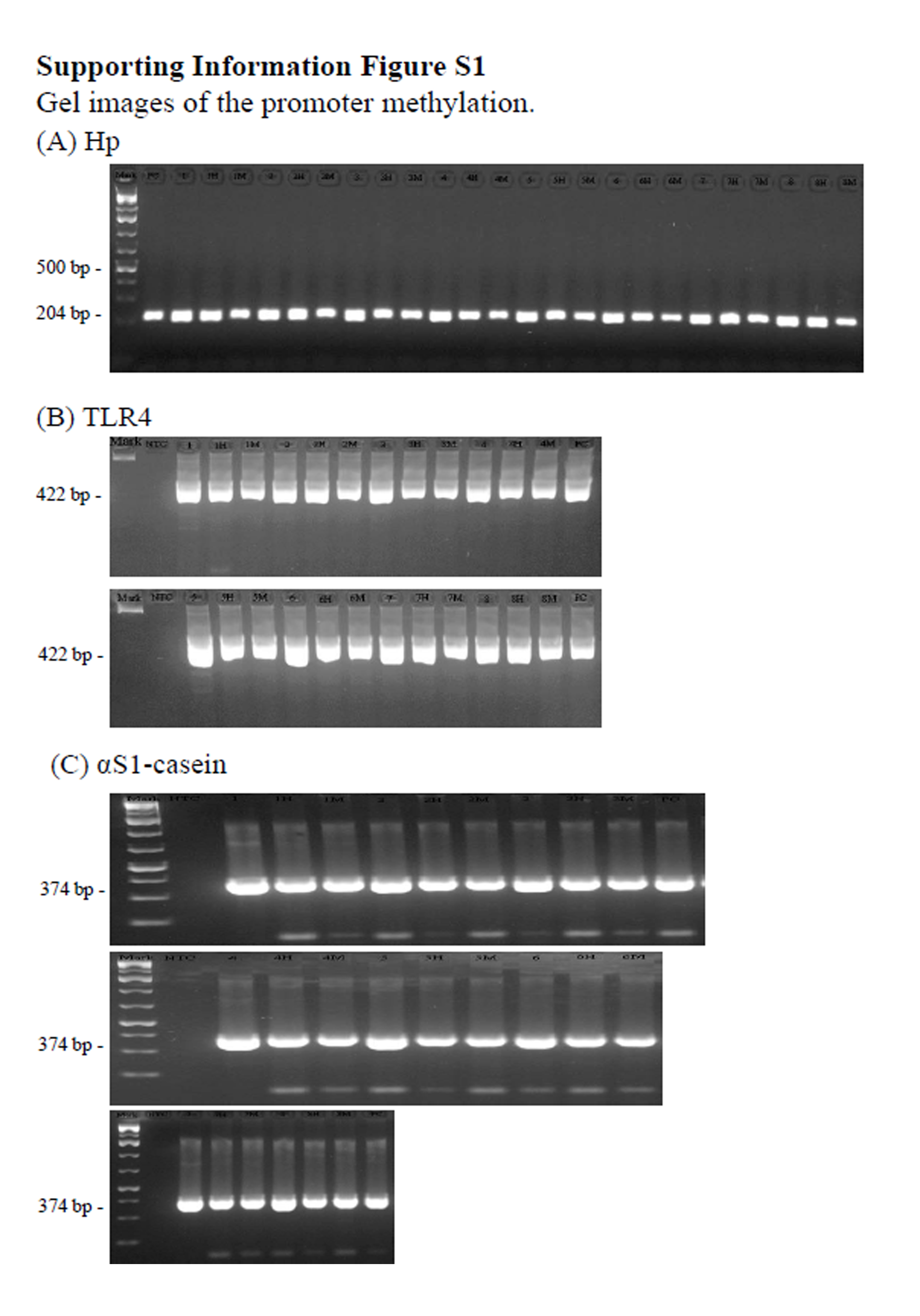

Supplement: S1 Fig — (TIF) [file pone.0123942.s001.tif]
