# Supplementary material for: Epigenetic Mechanisms Contribute to the Expression of Immune Related Genes in the Livers of Dairy Cows Fed a High Concentrate Diet
Source: PLoS One. 2015 Apr 10;10(4):e0123942. doi: 10.1371/journal.pone.0123942 (PMC4393131; doi:10.1371/journal.pone.0123942)
Supplement: S1 Table — (DOCX) [file pone.0123942.s002.docx]

**Supporting Information Table S1**

Primers for RT-qPCR.

| Gene | cDNA-specific primer | Forward primer | Reverse primer |
| --- | --- | --- | --- |
| TLR4 | CCACGGCCACCAGCTTCTG | GGACCCTTGCGTACAGGTTG | GGAAGCTGGAGAAGTTATGGC |
| LBP | CAAACTCTCAGGTACCGAACGT | GCAAGATCACTGGATTCTTGGA | AAAACAGGAAGTCCTTGTGGATC |
| IL-1A | GCAATGGCTTCCAGGTCATC | GGCCAAAGTCCCTGACCTCT | CTGCCACCATCACCACATTC |
| IL-1B | TGCCAGTCCTTGGGGTTATT | AACCGAGAAGTGGTGTTCTGC | TTGGGGTAGACTTTGGGGTCT |
| IL-6 | GGGAGCCCCAGCTACTTCAT | GGAGGAAAAGGACGGATGCT | GGTCAGTGTTTGTGGCTGGA |
| TNF-α | CTGTGAGTAGATGAGGTAAAGC | CTTCTGCCTGCTGCACTTCG | GAGTTGATGTCGGCTACAACG |
| IL-8 | CATGGAACAATGTACATGCGAC | CCTCTTGTTCAATATGACTTCCA | GGCCCACTCTCAATAACTCTC |
| IL-10 | GTGGGAGCTGAGGTATCAGAG | GTGGAGAAGGTGAAGAGAGTC | CGTCATGGAGTCTAGTAGAGTC |
| CCL5 | AGTTGGCGCAAGTTCAGGTT | TCCCCATATGCCTCGGAC | TCGCACCCACTTCTTCTCTG |
| CCL20 | TTCCATCCCAAAAAGCATCC | CAGCAAGTCAGAAGCAAGCAA | CCCACTTCTTCTTTGGATCTGC |
| SAA3 | GCCAGCAGGTCTGAAGTGG | CTTTCCACGGGCATCATTTT | CTTCGGGCAGCGTCATAGTT |
| Hp | GGCATCCAATGAGCCACCGAT | ACAAGGACCATTGGACAGCAACT | ACTGCCTCACATTCAGGGAGT |
| αS1-casein | TTTTTTTTTTTTTTTTTTTTN | CTTTTCAGACAATTCTACCAGCT | AATTCACTTGACTCCTCACCAC |
