# Supplementary material for: Epigenetic Mechanisms Contribute to the Expression of Immune Related Genes in the Livers of Dairy Cows Fed a High Concentrate Diet
Source: PLoS One. 2015 Apr 10;10(4):e0123942. doi: 10.1371/journal.pone.0123942 (PMC4393131; doi:10.1371/journal.pone.0123942)
Supplement: S2 Table — (DOCX) [file pone.0123942.s003.docx]

**Supporting Information Table S2**

Primers for CHART-PCR.

| Gene | Forward primer | Reverse primer | Length (bp) |
| --- | --- | --- | --- |
| TLR4 | CAGTTGTAGCTTGCAGGCTC | TTATAGTCCAACTCTCAACATCC | 422 |
| LBP | CAGAAAAGCAGAGAGTGGCTAG | GCCTCCTATTGGGAAACAGGT | 211 |
| Hp | CATTTTACTGATTTCAGGTTGGA | CAAGACTTGGGGAGGAACCGT | 223 |
| SAA3 | GGAGTGAGACTAGAAACGGG | GTGAAGCTGAGCTGCCTGTG | 269 |
| αS1-casein | AACAATCCATGACCATCCTGAC | AGGAAAGGAGTCCTGAAAGATG | 386 |
