# Supplementary material for: Epigenetic Mechanisms Contribute to the Expression of Immune Related Genes in the Livers of Dairy Cows Fed a High Concentrate Diet
Source: PLoS One. 2015 Apr 10;10(4):e0123942. doi: 10.1371/journal.pone.0123942 (PMC4393131; doi:10.1371/journal.pone.0123942)
Supplement: S3 Table — (DOCX) [file pone.0123942.s004.docx]

**Supporting Information Table S3**

Primers for methylation analysis.

| Gene | Forward primer | Reverse primer | Length |
| --- | --- | --- | --- |
| TLR4 | CAGTTGTAGCTTGCAGGCTC | TTATAGTCCAACTCTCAACATCC | 422 |
| LBP | CCTTGGCTATTTAAGGGAGC | GTCGGTGATCCTGACGACAAG | 180 |
| Hp | GCAGTTACCAACAGTTAGAAATG | GGTGCTGTCATATGCATATCTG | 204 |
| SAA3 | GGAGTGAGACTAGAAACGGG | GTGAAGCTGAGCTGCCTGTG | 269 |
| αS1-casein | CTGCCAGGTTCCTCTGTCC | CTGTTCAGGCTGTCCACTTTG | 374 |
